# Doping Polypyrrole Films with 4-N-Pentylphenylboronic Acid to Enhance Affinity towards Bacteria and Dopamine

**DOI:** 10.1371/journal.pone.0166548

**Published:** 2016-11-22

**Authors:** Mohsen Golabi, Laurence Padiolleau, Xi Chen, Mohammad Javad Jafari, Elham Sheikhzadeh, Anthony P. F. Turner, Edwin W. H. Jager, Valerio Beni

**Affiliations:** 1 Biosensors and Bioelectronics Centre, Department of Physics, Chemistry and Biology (IFM), Linköping University, Linköping, Sweden; 2 Cranfield Health, Cranfield University, Cranfield, United Kingdom; 3 School of Engineering, Physics and Mathematics, University of Dundee, Dundee, United Kingdom; 4 Deptartment of Physics, Chemistry and Biology (IFM), Linköping University, Linköping, Sweden; 5 Department of Chemistry, Faculty of Science, Ferdowsi University of Mashhad, Mashhad, Iran; Institute of Materials Science, GERMANY

## Abstract

Here we demonstrate the use of a functional dopant as a fast and simple way to tune the chemical affinity and selectivity of polypyrrole films. More specifically, a boronic-functionalised dopant, 4-N-Pentylphenylboronic Acid (PBA), was used to provide to polypyrrole films with enhanced affinity towards diols. In order to prove the proposed concept, two model systems were explored: (i) the capture and the electrochemical detection of dopamine and (ii) the adhesion of bacteria onto surfaces. The chemisensor, based on overoxidised polypyrrole boronic doped film, was shown to have the ability to capture and retain dopamine, thus improving its detection; furthermore the chemisensor showed better sensitivity in comparison with overoxidised perchlorate doped films. The adhesion of bacteria, *Deinococcus proteolyticus*, *Escherichia coli*, *Streptococcus pneumoniae* and *Klebsiella pneumoniae*, onto the boric doped polypyrrole film was also tested. The presence of the boronic group in the polypyrrole film was shown to favour the adhesion of sugar-rich bacterial cells when compared with a control film (Dodecyl benzenesulfonate (DBS) doped film) with similar morphological and physical properties. The presented single step synthesis approach is simple and fast, does not require the development and synthesis of functional monomers, and can be easily expanded to the electrochemical, and possibly chemical, fabrication of novel functional surfaces and interfaces with inherent pre-defined sensing and chemical properties.

## Introduction

Specificity is a key aspect that needs to be addressed in the development of sensors and/or functional surfaces. This can be enhanced by tuning the bio/chemical properties of the sensing interface via the addition of, for example, biomolecules (e.g. antibodies)[1, 2] or chemical ligands.[3, 4] Although this strategy has been widely explored, the methods used to modify the surface are often complex and time consuming requiring, for example, synthesis of novel functional monomers and/or a sequence of activation/conjugation steps. Therefore an easier, faster and more versatile approach to modify the surface or interface is needed.

Conducting polymers (CP) have been widely studied as sensor materials, due to their chemical, electrical and mechanical characteristics and to their ability to serve as both electronic and ionic charge carriers [5–8]. Among them polypyrrole (PPy) has been widely explored in cell biology for the development of functional surfaces for stimulation or recording of cells [9, 10], for controlling cell adhesion and proliferation of cells [11–14], for controlled drug release [15, 16], to mechanically stimulate single cells [17] and to serve as a sensing surface [18–20]. In order to improve the applicability of polymeric films in bio/chemo-sensors, active surface modification of their chemical/biochemical recognition ability and selectivity is needed. This has been achieved by pursuing two main routes; (i) the use of modified monomers (i.e. with COOH groups) followed, if required, by further functionalisation (i.e. antibodies or DNA) [19, 21, 22], or by (ii) the introduction of various counter ions (dopants) during the polymerisation step. The introduction of functional groups by co-polymerisation of pyrrole and modified pyrrole from a mixed monomer solution is challenging and often possible only under very specific conditions (e.g. a limited range of useful ratios between the different monomers) [23]. The use of dopants, including biomolecules [22, 24] or nano-materials [25, 26], has been shown to be a more advantageous and easier alternative to control the physical and chemical properties (i.e. thickness, roughness, conductivity and hydrophobicity) [14, 27–33] to introduce electrocatalytic properties [3] or to change the chemical properties of the PPy films by providing them with ion exchange ability or electrostatic perm-selectivity [34, 35].

In the work reported herein, we demonstrate the use of a boronic-functionalised dopant, 4-N-Pentylphenylboronic Acid (PBA), for the development of a polypyrrole-based surfaces with tuned affinity towards cis-diols. The fabricated films were shown to allow the retention and sensitive detection of dopamine and to have the ability to modulate bacteria adhesion.

Dopamine is a major neurotransmitter that is involved in neuroglial diseases such as parkinsonism, epilepsy and senile dementia [36] and is one of the main players in the molecular regulation of physical events such as pain and pleasure [37]. Dopamine detection is very challenging, not only due to its low concentration in biological fluids (tens of nM to low μM) but, even more importantly, because of the presence of other electroactive molecules (i.e. ascorbic acid and acetaminophen) at concentrations up to few hundreds time higher [38]. Although integrated catalysts, as such as CNTs, graphene [26, 39–41] and metal nanoparticles [42] in PPy films have been a popular route for the development of sensitive dopamine sensors, few examples of tuning the chemical properties of the films via the use of dopants can be found in literature. Harley et al. investigated the use of sulfonated *β*- cyclodextrin (due to its size/electrostatic affinity with dopamine) as dopant in the preparation of selective dopamine chemisensor [43]. Gholivand et al. [35] took advantage of the charge properties of aszophloxine doped overoxidesed polypyrrole film to perform the selective detection of dopamine over acetaminophen and ascorbic acid. [44] Jang et al. used tannic acid to dope PPy film.

Boronic acid is well-known to interact with cis-diol groups forming cyclic esters. This interaction has been explored for the development of electrochemical [45, 46] and optical sensors for the detection of saccharides, catechol [47, 48] and bacteria [49]. The interaction between the boronic group and dopamine has been used by several authors to develop specific dopamine sensors [50–52] based on the use, for example, of poly-aniline film containing boronic acid groups [50, 52]. Polymerisable boronic acid derivatives have also been used in the fabrication of dopamine specific molecular imprinted polymers (MIP) [45, 46]. Zhong et al. developed a strategy for the synthesis of boronic acid-modified polypyrrole monomer and applied it for fabrication of MIPs suitable for the detection of dopamine at the sub-μM level [53].

Selective, controllable and stable adhesion of bacteria/cells is of great interest in a variety of applications including: regulation of biofilm formation, bacterial differentiation [14] and in membrane bioreactors [54].

The bacterial outer membrane is a complex system differing widely in its morphology and chemical composition between types, species and strains, but is characterised by the presence on its surface of diol-rich molecules such as teichoic acids in Gram-positive and lipopolysaccharides (LPS) in Gram-negative bacteria. It should also be considered that some bacteria, depending on their growth phase, are coated with a polysaccharidic or proteic capsule. Clearly the richness of the cell wall membrane in polysacarides, makes them attractive targets for boronic-based manipulation and sensing.

Surfaces functionalised with boronic group have been already reported for the rapid capture of bacteria, yeast and human cells on surfaces [55–57]. Polsky *et al*. [57] developed, via electrochemical grafting of diazomium-modified boronic-acid containing molecules, a functional gold electrode for the on-demand release of previously captured yeast and mammalian macrophage cells. Kuralay *et al*. [56], modified a Ni/Pt microtube with a 3-aminophenylboronic acid polymer to build a “microrocket” for the capture and transport of yeast cells. Zhong *et al* [55]. Functionalised multiwalled carbon nanotubes with 3-aminophenylboronic acid and used them in the reusable cytosensors.

In this work we report a simple, fast and one step approach for the fabrication of PPy films with enhanced affinity towards diols. The proposed approach, based on the use of a commercially available functional dopant (4-N-pentylphenylboronic acid), has the advantage of not requiring the synthesis of functional monomers (often complex and time consuming) or the use of several fabrication steps; these aspects make the proposed concept highly versatile and appealing. In order to demonstrate the improved chemical properties of the film, its application for the selective retention and detection of dopamine and for the modulation of the adhesion of bacteria was explored.

## Experimental Section

### Materials

All reagents were of analytical grade and all were used without further purification. Dopamine hydrochloride (DA), ascorbic acid (AA), acetaminophen (AC), lithium chloride (LiClO_4_), sodium hydroxide (NaOH), 4-N-Pentylphenylboronic acid (PBA), Phenylboronic acid (BA), Dodecyl benzenesulfonate (DBS) and Tryptic soy broth (TSB) were purchased from Sigma-Aldrich (Stockholm, Sweden). Pyrrole was obtained from Fluka (Sweden), distilled and stored at -18°C. Structures of DA, AA, CA, PBA, BA and DBS are reported in Figure A in S1 File. Acetonitrile were purchased from Kebolab AB (Darmstadt, Germany). PBS (10 mM, pH 7.4) ready prepared tablets and Acetate buffer were supplied by Medicago AB (Uppsala, Sweden). All solutions were prepared in high purity water (18 MΩ) obtained from a Milli-Q RG system (Stockholm, Sweden).

*Deinococcus proteolyticus* and *Escherichia coli* were acquired from the University of Gothenburg Culture Collection (Sweden), while *Streptococcus pneumoniae* and *Klebsiella pneumoniae*, were obtained from Linköping University Hospital.

### Apparatus

All electrochemical measurements, film depositions and film overoxidation were carried out using either an EmStat2 electrochemical workstation together with PSTrace software (Palm Sense, The Netherlands) or an Ivium Stat.XR electrochemical analyser coupled with dedicated software (Ivium, Eindhoven, Netherlands).

Commercially available screen-printed carbon electrodes (4 mm disk; DS 110, DropSens S.L.; Oviedo-Asturias, Spain) and 5x10 mm Au coated Si-chips were used, respectively, for the fabrication of the dopamine and pathogen sensors. Gold substrates were produced in house by evaporating 2000 Å of Au and 100 Å of Ti on a Si wafer.

Evaluation of the chemical and morphological properties of the film was performed by contact angle measurements, ATR-FTIR measurements, SEM imaging and profilometric measurements.

Contact angle measurements were performed using a CAM 200 optical contact angle meter (KSV Instrument, Helsinki, Finland). ATR-FTIR measurements were carried out with a PIKE MIRacle ATR accessory with a diamond prism in a Vertex 70 spectrometer (Bruker) using a DTGS detector at room temperature under continuous purging of N_2_. IR spectra were acquired at 4 cm^-1^ resolution and 32 scans between 4000 and 800 cm^-1^. The Scanning Electron Microscopy (SEM) micrographs were taken using a Leo 1550 Gemini SEM operating at 3.0 keV. The thickness and roughness of the samples were determined using a Dektak 6M Profilometer (Veeco Instruments Inc., NY). The concentrations of the cultured bacteria were calculated using spectrophotometric measurements (600 nm) using a Shimadzu UV-1601 PC spectrophotometer.

To determine the adhesion level of bacterial strains to each surface, the samples were stained with ethidium bromide. Images of the samples’ surface were then taken using a ZEISS fluorescent microscope and analysed using open source image J software.

### Dopamine sensor

*Sensor fabrication*: Prior to the electrodeposition of the polypyrrole film the screen-printed carbon electrodes were subjected to an anodic activation (1.6 V vs Ag internal reference electrode, 90s in saturated aqueous solution of Na_2_CO_3_). Following exhaustive rinsing with MilliQ water and drying with N_2_, the working electrodes were covered with 80 μL of a water/acetonitrile (4:1 ratio) solution containing 75 mM of pyrrole monomer and different concentration of the dopant (4-N-pentylphenylboronic acid or LiClO_4_) ranging from 1 and 10 mM; electrodeposition of the recognition layer was performed via cyclic voltammetry (potential interval -0.2/0.7 V, scan rate 0.02 Vs^-1^, number of scans: 20). Thereafter, the PPy film was overoxidised by applying 1.1 V for 180 s in 100 mM NaOH solution. Cyclic voltammetry (CV) was chosen as the fabrication method to allow better comparison of the sensor performance with the literature [35].

**Fig 1:**
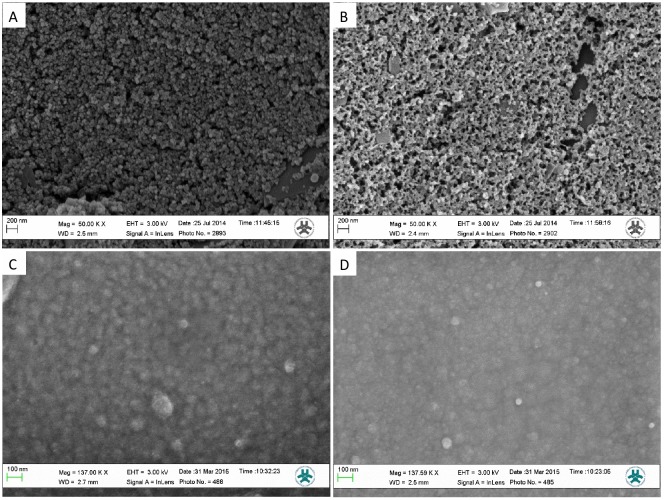
SEM images of different PPy films: A) PPy film on carbon electrode using PBA 1 mM as dopant; B) OxPPy film on carbon electrode using PBA 1 mM as dopant; C) PPy film on Au using PBA 1 mM as dopant; D) PPy film on Au using DBS 1 mM as dopant. All films were prepared from an acetonitrile water solution (1:4) containing 75 mM of Py and 1 mM of the dopant (PBA or DBS or ClO_4_^-^).

*Sensor characterisation and dopamine detection*: Characterisation of the developed sensors was performed either by CV (in all figures 1 scans are reported) or by amperometric measurements. Selectivity studies were performed by recording cyclic voltammograms (potential interval -0.2/0.6 V; scan rate 0.02 Vs^-1^) in the presence of different concentrations (between 0.5 μM and 2 mM) of dopamine or interfering molecules (ascorbic acid and acetaminophen). The ability of the 4-N-pentylphenylboronic acid-doped polymeric film to retain dopamine was demonstrated by exposing the sensor to a 250 μM solution of dopamine in PBS buffer for a well-defined time followed by measurement (by cyclic voltammetry) in fresh PBS solution. Calibration curves were recorded in PBS solution, under gentle stirring, using amperometric techniques (applied potential 0.4 V vs internal Ag reference). Regeneration of the sensor surface, when needed, was performed by acid washing (10 mM acetic acid solution pH 2.75).

### Surface for bacteria adhesion

#### Surface fabrication

Prior to the electrodeposition of the polymeric films, the Au chips were cleaned using a RCA1 solution (5:1:1 mixture of D.I water, ammonia 25% and hydrogen peroxide 28%, respectively, for 10 min at 85°C) and kept, prior to use, in de-ionised water. Electrochemical polymerisation was performed in a single compartment cell using the Au chip as the working electrode (active area ca. 0.25 cm^2^), a Pt mesh as the counter electrode and Ag/AgCl as the reference electrode. Polymerisation was performed from a 75 M solution of pyrrole and 1 mM of PBA or DBS (dopant) in a 1:4 acetonitrile: water solvent media via the potentiostatic method (0.7 V vs Ag/AgCl reference until charge density reached 5 mC cm^-2^). Overall thickness of the film was found to be ~1500 nm, (Table 1). The prepared films were washed with deionised water, dried with nitrogen and stored at 4°C until use. Potentiostatic synthesis was chosen because this allows an excellent tuning of the film morphology [14, 33]; thus excluding the effect of differences in surface morphology on the adhesion on the PBA and DBS surfaces.

**Table 1:** Physicochemical properties of polypyrrole-doped polymer. All films were prepared from an acetonitrile water solution (1:4) containing 75 mM of Py and 1 mM of the dopant (PBA or DBS).

Type of polymer	Thickness (nm)	Roughness (nm)	Water Contact Angle
Polypyrrole doped with DBS (PPy-DBS)	1563 ± 145	277 ± 70	73° ± 5°
Polypyrrole doped with PBA (PPy-PBA)	1442 ± 76	175 ± 43	74° ± 2°

#### Film characterisation

The chemical/physical properties of the prepared film were evaluated via different techniques including contact angle and ATR-FTIR measurements. In the contact angle measurements, one drop (10 μl) of fresh Milli Q water was placed on the polymer surface and an image was recorded. The contact angle, computed by the accompanying software, was recorded as the measured contact angle. ATR-FTIR measurements on bacteria suspensions were used for studying their interaction with boronic groups and were performed according to the following protocol: *E*.*coli* and *D*.*proteolyticus* were cultured overnight as described in the bacterial experiment section. Following their recollection by centrifugation (4000 g, 5 min) these were re-suspend in phenylboronic acid (BA) (100mg/ml) for 5 min at room temperature with gentle shaking. After treatment, the cells were collected by centrifugation and washed in PBS three times to remove non-bounded BA. The cell pellet was then used for the ATR-FTIR test. The PPy films, doped with PBA and DBS, electrodeposited on Au chips were used for ATR-FTIR characterisation. Morphological studies of film thickness and roughness, were performed via SEM and profilometric measurements using the Dektak Profilometer, represented in Fig 1 and Table 1.

#### Bacterial experiments

Bacterial strains were grown in TSB for 18 hrs, at 37°C and 170 rpm and washed three time in PBS prior to use. After the last wash, the concentration of bacterial cell was adjusted to 5×10^7^ cfu/ml. The optical density equal to 5×10^7^ cfu/ml for each bacterial strain was previously determined by the standard plate count method. Adhesion studies were performed by placing the prepared surfaces in contact with a 5×10^7^ cfu/ml bacterial cell suspension of the specific strain. PPy film samples were placed in each well of a 6-well microtiter-plate together with 7.5 mL bacterial cell suspension (in saline solution) and incubated at 70 rpm for 30 minutes at 37°C. The samples were then gently rinsed 3 times with PBS to remove non- or weakly adhered cells. The attached cells were then stained using ethidium bromide (0.05%, 45 min) and imaged with a fluorescent microscope. For each sample, 10 images were taken across the entire surface; these were analysed using the image-analysis software program ImageJ. The reported number of attached cells on each polymer is the average of ten slides for each of three repeat samples.

## Results and Discussion

In order to introduce boronic functionalities into the polypyrrole films 4-N-pentylphenylboronic acid (PBA) was used as dopant. From among the different possible boronic containing dopants, PBA was chosen because of its structure; its long hydrophobic chain and its aromatic head were envisaged to facilitate a stable incorporation in the polymeric film via hydrophobic and π-π interactions. Although the hydrophobic character was beneficial to achieve a stable incorporation in the film, at the same time this was also a serious limitation as it was poorly soluble in water. As a result, film preparation was only possible from water/acetonitrile solutions (4:1) containing low concentration of PBA (below 10 mM). Based on the results of screening tests (data not shown) it was decided to use 1 mM as the concentration of PBA in the electrodeposition solution. Subsequently, all films were prepared using 1 mM of the dopant (DBS, PBA or ClO_4_^-^).

### Dopamine sensor development

Preliminary studies (data not shown) indicated that a potential window between -0.2 and 0.7 V was the most suitable electro-polymerisation window for polymer fabrication via CV; a lower upper-end potential (0.4 V) resulted in non-polymeric film deposition and a higher upper-end potential (1 V) resulted in the formation of partially overoxidised and thick films. Different PPy films, containing PBA or ClO_4_^-^ (1 mM) as dopant, were fabricated, overoxidised (if required) and their sensing performances compared. In Fig 1, typical SEM images for non-overoxidised (Fig 1A) and overoxidised (Fig 1B) PPy-PBA films are presented. As can be seen from Fig 1A, the use of PBA as a dopant did not have any significant influence on the morphology of the PPy film, which had a uniform nodular morphology typical of PPy films [58]. Significant changes in the overall morphology of the PPy film were recorded following its overoxidation (Fig 1B); the films became less uniform presenting linked granules and a much more porous structure [35]. Overoxidation of the film was used to minimise the selectivity problems associated with the intrinsic conductivity of conductive polymers [59] by converting the PPy in an inert semipermeable negatively-charged film [35]. Demonstration of the effect of overoxidation was performed by voltammetric studies, using dopamine, ascorbic acid and acetaminophen (Figure B in S1 File). A significant change in the electrochemical behaviour of the molecule investigated can be clearly seen in Figure B in S1 File; the introduction of PPy-PBA film onto the carbon surface resulted in the complete suppression of the signal associated with ascorbic acid (due to electrostatic repulsion) and the significant improvement in the dopamine response (due to electrostatic and chemical interaction with the PBA-PPy film). The overoxidation of the film resulted in a further improvement in the electrochemical response towards dopamine and in an ever more significant improvement of the electrochemical response for ascorbic acid clearly demonstrating, as expected, significant changes in the electrochemical and morphological properties of the film. The obtained film was now a strongly porous and negatively charged perm-selective membrane with significant affinity for positively charged molecules (dopamine and ascorbic acide) as previously reported [35], but with also an expected added affinity towards diol-containing molecules (DA) due to the presence of the boronic group in the film [60].

**Fig 2:**
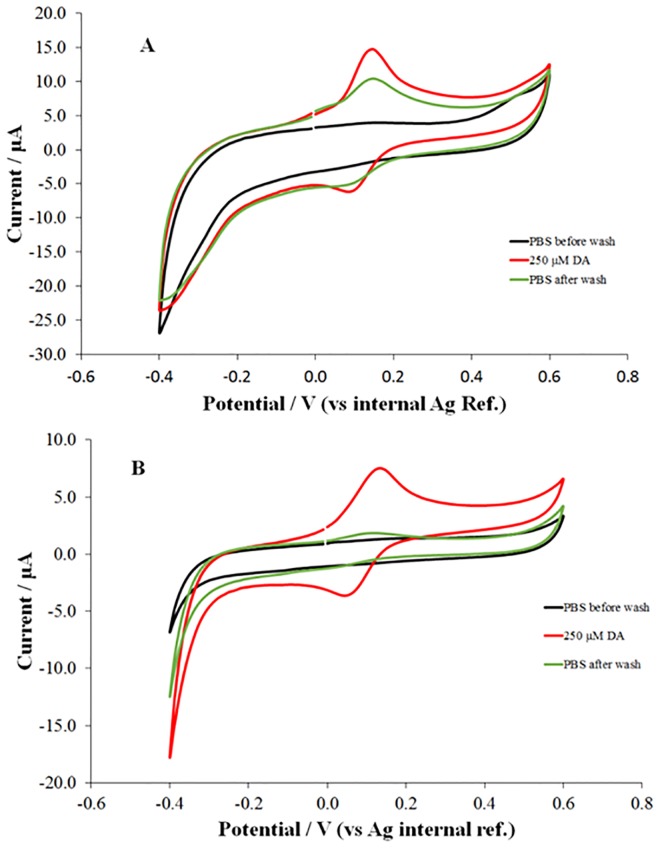
Retention experiment at A) OxPPy-PBA and B) OxPPy-ClO_4_ film electrodes. Both films prepared using 1 mM of the dopant. All films were prepared from an acetonitrile water solution (1:4) containing 75 mM of Py and 1 mM of the dopant (PBA or ClO_4_^-^).

Having demonstrated the effectiveness of the overoxidation process, the ability of the film to retain dopamine, via the boronic group/diol interaction, was investigated. In order to evaluate this, two films using PBA or ClO_4_^-^ as the dopant were fabricated, overoxidised and exposed for 5 minutes to a 250 μM solution of dopamine in PBS. PBS pH 7.4 was chosen as working solution, since it resembled the pH conditions of physiological fluids; even though this pH is not ideal to maximise boronic group dopamine interactions, significant complexation can still be expected [61]. As can be seen from Fig 2, a well-defined redox response was recorded for both films. Following washing of the electrode with PBS, new voltammetric measurements were recorded in PBS (pH 7.4) in the absence of dopamine to evaluate the ability of the developed film to capture/retain the analyte.

As can be seen in Fig 2A, in the case of OxPPy-PBA electrodes a clear electrochemical response was recorded following sensor washing (green line); on the other hand no significant electrochemical response was recorded when similar experiments were performed at the OxPPy- ClO_4_ film electrode (Fig 2B, green curve). The results of these experiments clearly demonstrated that the film has the ability to capture dopamine. Further demonstration of the retention ability of the OxPPy-PBA film was the significant reduction of the peak to peak separation when compared to the OxPPy-ClO_4_ film; peak to peak separations of ca. 60 mV were recorded in the case of the PBA film compared to ca. 85 mV recorded at the ClO_4_ film. This result seems to indicate, in the case of the OxPPy-PBA film, the presence of a surface confined contribution to the overall electrochemical response of the sensor.

In order to demonstrate that the recorded effect was not due to a pure electrostatic interaction between the PBA and the positively charged dopamine, a similar experiment was performed using acetoaminophen, another positively charged molecule. The supporting information (Figure C in S1 File) shows that washing the electrode in PBS resulted in a complete loss of the electrochemical response, indicating no retention of the acetoaminophem in the OxPPy-PBA film.

Having demonstrated the ability of the developed surface to capture dopamine, the time domain of the process was studied. Prior to this a protocol for the regeneration of the surface, based on acid washing (10 mM acetic acid solution pH 2.75), was developed. The boronate ester is known to be stable in an alkaline aqueous, but to be reversed in an acidic environment [62, 63]. As expected, washing in acetic acid solution (for 30 min) resulted in the dissociation of the boronic group/dopamine complex [61], suppressing the redox signal of dopamine (Figure D in S1 File) even after the sensor surface had been exposed to high concentration of dopamine (2 mM). Using the optimised regeneration protocol, a study of the kinetics of the capture process was performed by incubating OxPPy-PBA electrodes with a 250 μM solution of dopamine for different times, followed by electrochemical measurements. In between the two consecutive measurements, the electrode was washed in acetic acid solution for 30 min. in order to remove the previously captured dopamine.

**Fig 3:**
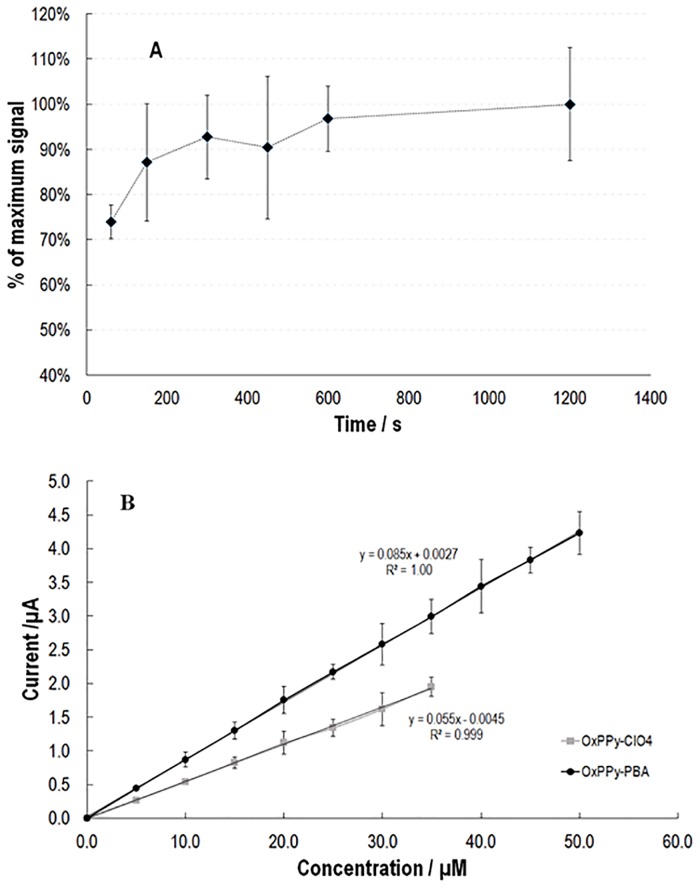
A) Assessment of the time for binding between the PPy-PBA film and dopamine B) Amperometric detection—Variation in current response as a function of dopamine concentration on OxPPy-ClO_4_ electrode and OxPPy-PBA modified electrode. All films were prepared from an acetonitrile water solution (1:4) containing 75 mM of Py and 1 mM of the dopant (PBA or ClO_4_^-^).

As can be seen from Fig 3A, the capture process is fast, reaching a response of between 80% and 90% of those recorded after 1200 s (maximum value obtained) in 120 s. No other reference with regards to the capture dynamics of similar systems could be found in the literature. Next, calibration curves for the OxPPy-PBA and OxPPy-ClO_4_ were determined using amperometry (Fig 3B). In order to take the capturing kinetics of the sensing surface into consideration, 120 s were left between two consecutive additions of the analyte. A typical result for an amperometric experiment for the detection of dopamine is presented in Figure E in S1 File.

The calibration curve recorded with the OxPPy-PBA film electrode was linear between 5 and 50 μM and showed higher sensitivity (ca. 1.5 times) when compared to OxPPy-ClO_4_; despite this increase in sensitivity no significant improvement in the limit of detection (4.1 μM; 3 times SD background signal) was achieved.

### Bacteria adhesion studies

Bacteria cell walls are very complex structures and their composition and morphology can vary significantly (e.g. Gram-positive and Gram-negative) especially with respect to the polysaccharides, proteins and lipids that are their main components. Clearly the richness in polysacarides, and subsequently diols, at the interface between the cell wall and the environment makes bacteria an attractive target for investigating the adhesion modulation due to the presence of boronic group in the polymeric matrix. It is well-known that the physicochemical properties, for example roughness and wettability of the film, can influence bacterial adhesion [64–66]. In order to minimise the influence of the morphological and physical properties of the films on the bacteria, adhesion films (control and test film) with comparable wettability and roughness were developed by controlling the fabrication process (polymerisation conditions) and the nature of the dopants [28, 67, 68]. DBS was found to be the most suitable dopant to be used in the control surface (PPy-DBS film). In Table 1 some of the physical properties of the two PPy films (PPy-PBA and PPy-DBS), are reported.

The morphological similarities between the two films were also confirmed from SEM images (Fig 1C and 1D). ATR-FTIR measurements were performed to confirm the incorporation of the boronic group in the prepared PPy-PBA film. A PPy-ClO_4_ film was used as control film instead of PPy-DBS; to avoid interference of DBS in the 3000–3600 cm^-1^ region.

**Fig 4:**
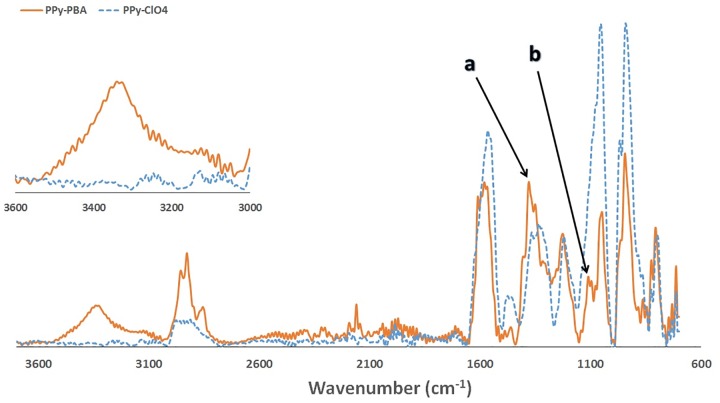
ATR-FTIR spectrum of PPy doped with PBA and ClO4^-^. All films were prepared from an acetonitrile water solution (1:4) containing 75 mM of Py and 1 mM of the dopant (PBA or ClO_4_^-^).

Fig 4 shows that the PPy-PBA film has a broad band appearing around 3350 cm^-1^; which can be attributed to the O-H vibration in PBA molecules and to the associated hydrogen bonding [69]. The B-O asymmetric stretch around 1350 cm-1 (a) and B-C stretching around 1080 cm-1 (b) are also shown [70].

**Fig 5:**
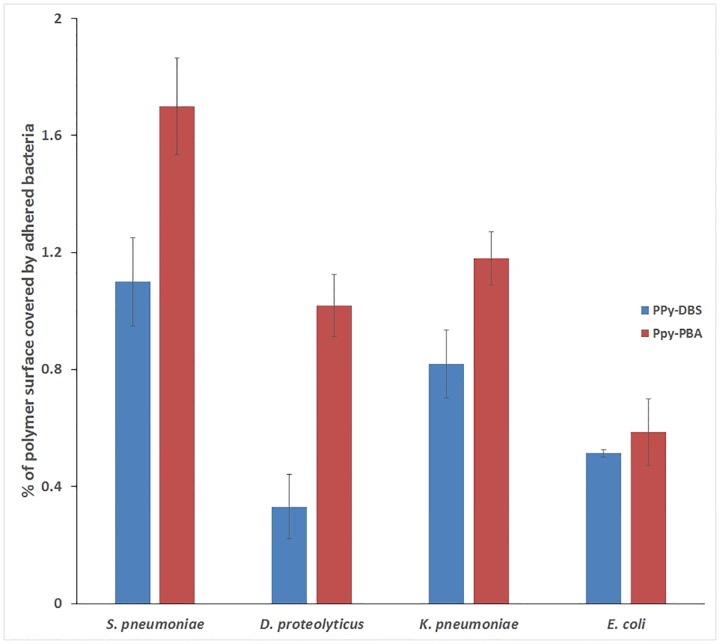
The effects of different doping of PPy on bacterial adhesion to the surface. DBS and PBA doped PPy films were exposed by different bacterial suspension (7.5 ml of 5 × 10^7^ cfu/ml, 30 min). The numbers of bacteria were determined by fluorescent dye staining and imaging. Error bars indicate standard deviations. All films were prepared from an acetonitrile water solution (1:4) containing 75 mM of Py and 1 mM of the dopant (PBA or DBS).

Fig 5 shows the adhesion of four different bacteria strains on DBS and PBA doped PPy. As can be seen from this Figure, the presence of the boronic group increased the adhesion of three out of the four bacteria.

Given the similar surface physiochemical properties (Table 1) of the two films, the increased adhesion of bacteria to PPy-PBA can be attributed to the chemical properties introduced by the dopant.

In our previous works we showed the effect of surface properties, like roughness and hydrophobicity, on the adhesion of different bacteria [14, 33]. Perera-Costa et al. [71] reported that bacterial adhesion can be promoted by 30−45% by micro patterning of surface and that this is a general phenomenon which occurs in both Gram-positive and Gram-negative cells with spherical or rod shapes. Here we introduced a new concept, based on taking advantage of the chemical composition of bacterial surfaces, to improve bacterial adhesion selectivity by taking advantage of the chemical interaction between chemical groups in the film (boronic group from PBA dopant) and the sugar-rich structure of the bacteria outer membrane. Significantly, the introduction of functional groups to the PPy film differentially increased the bacterial adhesion as a function of the bacteria tested.

Significant increase in adhesion (up to 3 times) was recorded for *D*.*proteolyticus*. This bacterium, despite being Gram-positive, is characterised by a cell wall similar to those of Gram-negative bacteria. *Deinococcus* is characterised by an outer layer consisting of regularly packed hexagonal protein subunits, a dense carbohydrate coat structure have also been reported in some strains [72]. This coating structure could be responsible for the increased adhesion to the PPy-PBA film in comparison to the PPy-DBS film. *S*. *pneumoniae*, a Gram-positive bacterium, is characterised by an outer membrane rich in sugar which could be the reason for the high adhesion of these bacterial cells to the PPy-PBA film. Interestingly, *K*. *pneumoniae* and *E*. *Coli*, both being Gram-negative, showed significantly different behaviours, with an increase in adhesion for the first and an unchanged adhesion for the latter, when compared to PPy-DBS. These results can be ascribed to the increased complexity and variability of the cell wall of Gram-negative bacteria.

In order to further demonstrate that the boronic group was involved in the bacteria/surface interaction, the adhesion experiments were repeated with bacterial cells having the diol-groups chemically “blocked”; as this was the most feasible approach to perform adhesion experiment with diol-free bacteria cells. Chemical blocking of the diol groups was achieved by pre-incubating the bacteria with phenyl boronic acid (BA) (100 mg/ml) for 5 min. The use of BA instead of PBA was dictated by the low solubility of the latter; BA was seen as a good alternative allowing a higher saturation in aqueous solution without changing the recognition region (the phenyl boronic ring).

**Fig 6:**
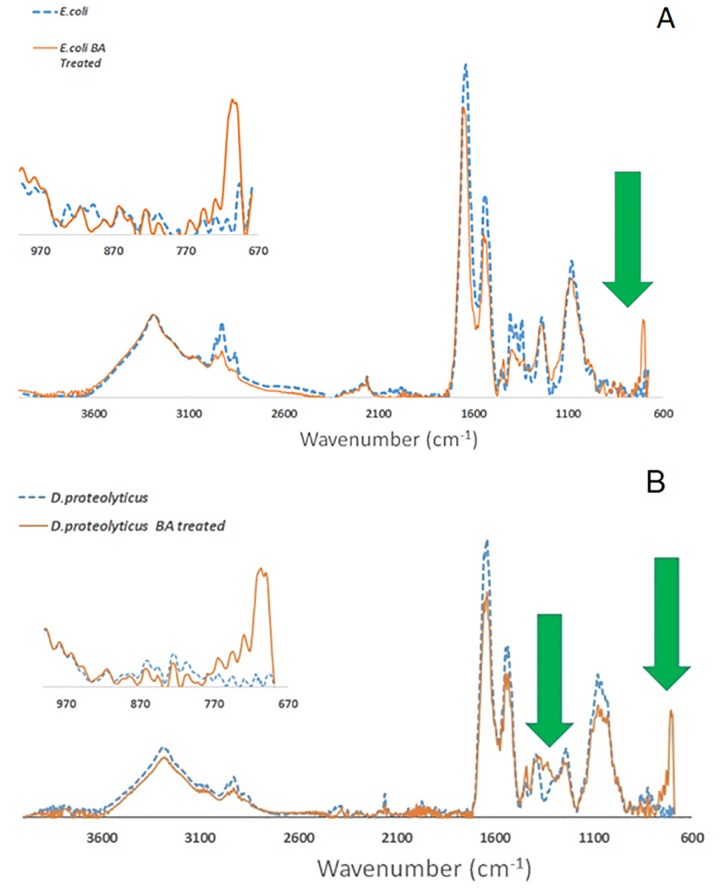
ATR-FTIR spectrum of E.coli A) and D.proteolyticus B) before and after treatment with BA.

*D*.*proteolyticus* and *E*.*coli*, the two bacteria which showed the most and least difference in adhesion, were used for this set of experiments. In Fig 6(A) and 6(B) the ATR-FTIR spectra recorded for the two bacteria before and after treatment with BA are compared. As can be seen, a sharp peak around 700 cm^-1^ appeared in the spectra (inset Fig 6A and 6B) following treatment with BA, which might be due to the C-H out-of-plane vibration and ring deformation of the phenyl unit of the BA molecule. The B-O asymmetric stretch around 1350 cm^-1^ also appeared following treatment of *D*. *proteolyticus* with BA (Fig 6B).

This typical signal for the boronic group was recorded in the pellets collected from both *E*.*coli* and *D*.*proteolyticus*. Although no differences in the adhesion of *E*.*coli* were recorded between PPy-PBA and PPy-DBS (Fig 5), the ATR-FTIR results in Fig 6A revealed an interaction between BA and the bacterial cells. This result could be explained in two ways: a) assimilation/adsorption of BA by the bacteria [73]; or b) BA had reacted with the sugars units present in the inner layer of the bacteria membrane that makes them not available for interaction with the boronic group available in the PPy-PBA film.

**Fig 7:**
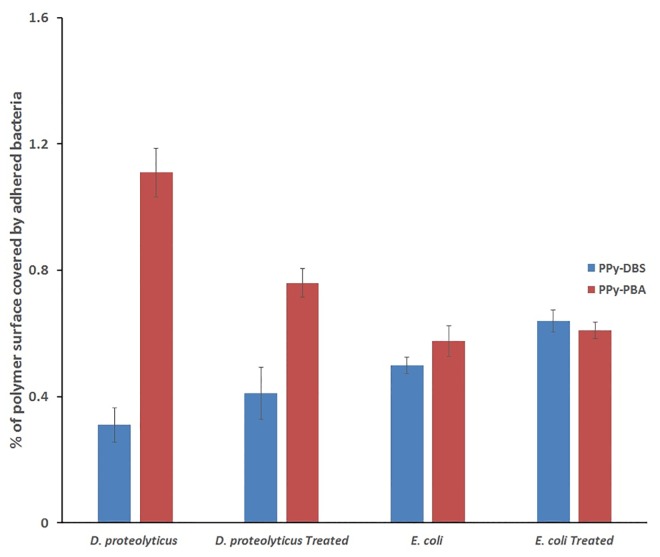
The effects of BA treatment on bacterial adhesion to the functionalised surfaces. *D*.*proteolyticus* and *E*.*coli* were pre-treated with BA (100 mgr/ml, 5min), collected by centrifugation, re-suspended in PBS and then exposed to the polymer surfaces. Untreated bacterial cells under the same conditions (7.5 ml of 5 × 10^7^ cfu/ml, 30 min) were used as a control test. The numbers of bacteria were determined by fluorescent dye staining and imaging. Error bars indicate standard deviations. All films were prepared from an acetonitrile water solution (1:4) containing 75 mM of Py and 1 mM of the dopant (PBA or DBS).

Comparison of the adhesion experiments performed with treated and untreated *E*.*coli* and *D*.*proteolyticus* (Fig 7), clearly demonstrate the importance of the sugar boronic group interaction in modulating the interaction between the polymer surface and the bacteria.

As can be seen from Fig 7, the adhesion of *D*.*proteolyticus* onto the PPy-PBA surface was decreased when bacteria cells pre-treated with BA were used. This result seems to indicate that the use of BA blocked cis-diol groups present at the outer surface of this bacteria, making them unavailable for interaction with the boronic groups present in the PPy-PBA film and thus interfering with the adhesion of the bacteria to the PPy-PBA film. In the case of the *E*.*coli*, no significant change in the adhesion was recorded before or after the treatment of the bacteria with BA. This result seems to indicate that in the case of *E*.*coli*, the cis-diols are not present at the outer shell of the bacteria being in this way not available for to interact with the boronic groups in the PPy-PBA film.

## Conclusions

A novel simple approach, based on the use of commercially available dopant, for the fabrication of polypyrrole films with enhanced affinity towards diols is presented. This enhanced affinity was achieved by the use of PBA (4-N-Pentylphenylboronic acid) as the dopant in the PPy films. The use of such a functional dopant allowed for easy, fast and efficient tuning of the sensing and bio-affinity properties of the PPy films. This has been demonstrated via the development of (i) a dopamine chemisensor and a (ii) surface with improved bacteria capturing capabilities.

The dopamine chemisensor, based on overoxidised PBA doped PPy film was shown, via incubation/washing experiments, to have the ability to chemically capture and retain dopamine. The capture process was fast (equilibrium reached after 5 min.), stable and chemically reversible. The chemisensor showed improved chemical affinity towards dopamine, with respect to sensitivity (8.5 10^−2^ μAμM^-1^ vs 5.5 10^−2^ μAμM^-1^) and dynamic range (5 to 50 μM vs 5 to 30 μM) when compared with perchlorate-doped film. However, no significant improvement in the detection limit of the sensor was observed and the limit of detection (4.1 μM) is significantly higher than other values reported in the literature (between 1 nM and 100 nM) for catalytic carbon-based nanomaterials (i.e. CNTs or graphene) as can be seen in Table A in S1 File in the supporting information.

Boronic-modified surfaces were also tested for the adhesion of *Deinococcus proteolyticus*, *Escherichia coli*, *Streptococcus pneumoniae* and *Klebsiella pneumoniae* bacterial cells. When compared with DBS doped film, which presented analogous chemical/physical properties, the PBA doped film showed increased adhesion of sugar rich bacterial cells and enhanced selectivity as compared with previous work [14, 33]. Furthermore these preliminary studies seem to indicate that the adhesion of the bacteria to the surface is not only dependent on the presence of the diol groups in the cell membrane of the bacteria, but also on their accessibility for interaction.

The use of PBA as a boronic functionalised dopant provides a simple and versatile approach to improve the chemical affinity of polypyrrole films towards diol-containing targets and has immediate application in the design of various sensors and may also be useful in bioreactor design.

The presented approach, based on the use of a commercially available dopant, is a simple and fast way of tuning the chemical properties of PPy films. This could be easily expanded to the electrochemical, and possibly chemical, fabrication of a variety of CP based functional surfaces and interfaces with improved sensing properties and chemical functionalities that could find applications in various areas such as environmental analysis (e.g. heavy metal analysis), microbiology (e.g. selective separation of bacteria) or biomedical analysis (e.g. cell patterning and/or chemical differentiation).

## Supporting Information

S1 File Figure A, Structure of some of the molecules used in the work. Figure B, CV experiments for PBS, DA, AA and AC recorded at bare carbon electrode, PPy-PBA modified electrode and OxPPy-PBA electrode. Cyclic voltammetry (scan rate 50 mV/s) in PBS in the presence of 250 μM of DA or AC. All films were prepared from an acetonitrile water solution (1:4) containing 75 mM of Py and 1 mM of the dopant (PBA). Figure C, Retention studies for AC at the OxPPy-PBA film. The film was prepared from an acetonitrile water solution (1:4) containing 75 mM of Py and 1 mM of the dopant (PBA). Figure D, Evaluation of the acetate solution washing on the retention of DA at the OxPPy-PBA film. The film was prepared from an acetonitrile water solution (1:4) containing 75 mM of Py and 1 mM of the dopant (PBA). Figure E, Typical amperometric calibration, in PBS, for DA at the OxPPy-PBA film. The film was prepared from an acetonitrile water solution (1:4) containing 75 mM of Py and 1 mM of the dopant (PBA). Figure F, Graphical abstract: Schematic representation of the use of boronic-modified dopant for the improved capture of bacteria (A) and of the concentration of dopamine (B). Table A, Comparison of proposed dopamine sensing surface with a selection of relevant literature.
(DOCX)
