# Supplementary material for: Doping Polypyrrole Films with 4-N-Pentylphenylboronic Acid to Enhance Affinity towards Bacteria and Dopamine
Source: PLoS One. 2016 Nov 22;11(11):e0166548. doi: 10.1371/journal.pone.0166548 (PMC5119770; doi:10.1371/journal.pone.0166548)
Supplement: S1 File — Figure A, Structure of some of the molecules used in the work. Figure B, CV experiments for PBS, DA, AA and AC recorded at bare carbon electrode, PPy-PBA modified electrode and OxPPy-PBA electrode. Cyclic voltammetry (scan rate 50 mV/s) in PBS in the presence of 250 μM of DA or AC. All films were prepared from an acetonitrile water solution (1:4) containing 75 mM of Py and 1 mM of the dopant (PBA). Figure C, Retention studies for AC at the OxPPy-PBA film. The film was prepared from an acetonitrile water solution (1:4) containing 75 mM of Py and 1 mM of the dopant (PBA). Figure D, Evaluation of the acetate solution washing on the retention of DA at the OxPPy-PBA film. The film was prepared from an acetonitrile water solution (1:4) containing 75 mM of Py and 1 mM of the dopant (PBA). Figure E, Typical amperometric calibration, in PBS, for DA at the OxPPy-PBA film. The film was prepared from an acetonitrile water solution (1:4) containing 75 mM of Py and 1 mM of the dopant (PBA). Figure F, Graphical abstract: Schematic representation of the use of boronic-modified dopant for the improved capture of bacteria (A) and of the concentration of dopamine (B). Table A, Comparison of proposed dopamine sensing surface with a selection of relevant literature. (DOCX) [file pone.0166548.s001.docx]

# Supporting Information

**Doping polypyrrole films with 4-N-pentylphenylboronic acid to enhance affinity towards bacteria and dopamine**

Mohsen Golabi^1^ , Laurence Padiolleau^1,2^ , Xi Chen^1,3^ , Elham Sheikhzadeh^1,4^ , Anthony P.F. Turner ^1^, Edwin W.H Jager ^1*^, Valerio Beni ^1, #a, *^


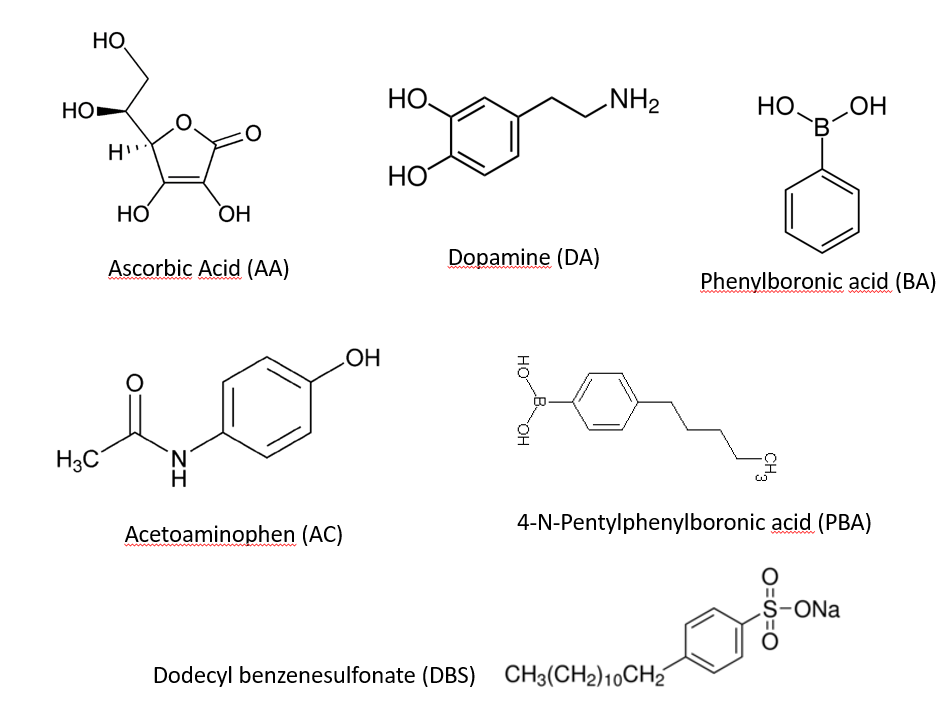


**Figure A.** Structure of some of the molecules used in the work.


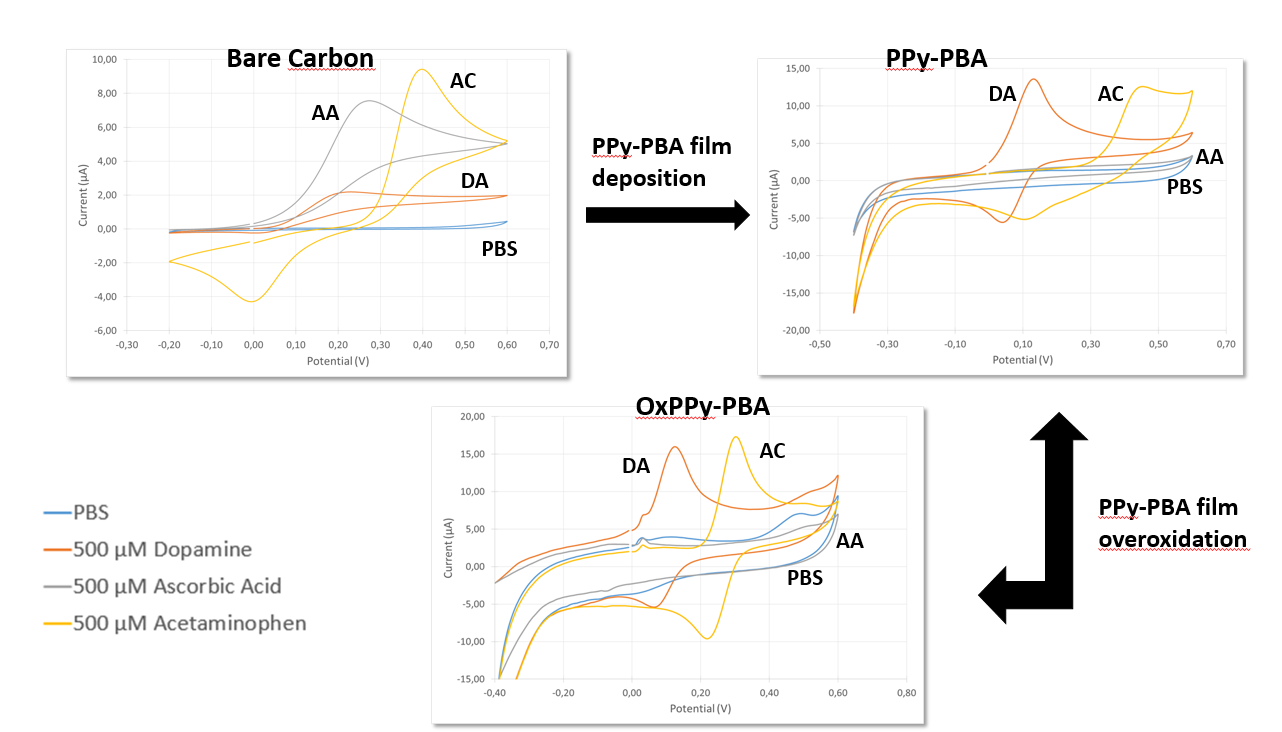


**Figure B.** CV experiments for PBS, DA, AA and AC recorded at bare carbon electrode, PPy-PBA modified electrode and OxPPy-PBA electrode. Cyclic voltammetry (scan rate 50 mV/s) in PBS in the presence of 250 µM of DA or AC. All films were prepared from an acetonitrile water solution (1:4) containing 75 mM of Py and 1 mM of the dopant (PBA).


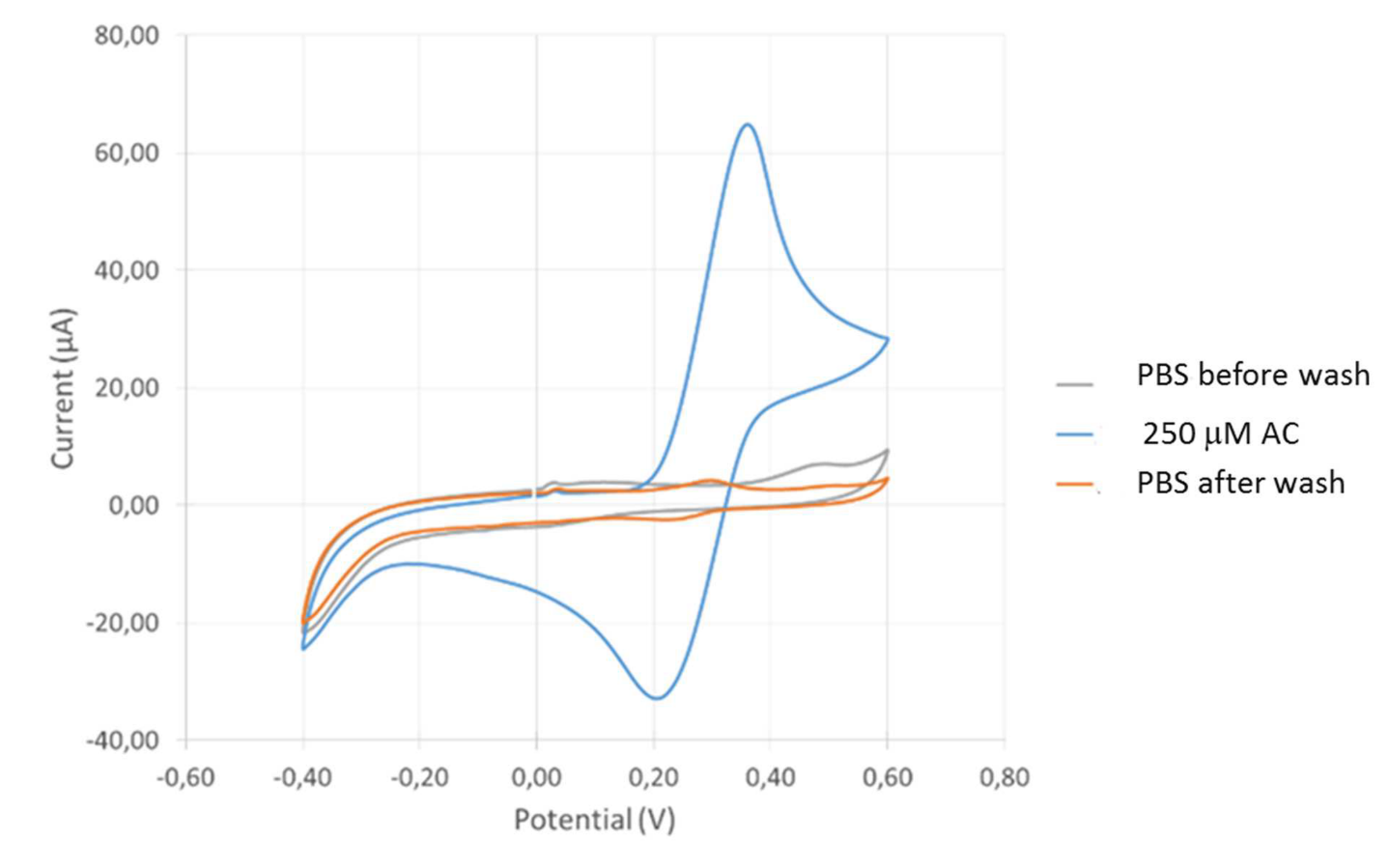


**Figure C.** Retention studies for AC at the OxPPy-PBA film. The film was prepared from an acetonitrile water solution (1:4) containing 75 mM of Py and 1 mM of the dopant (PBA).


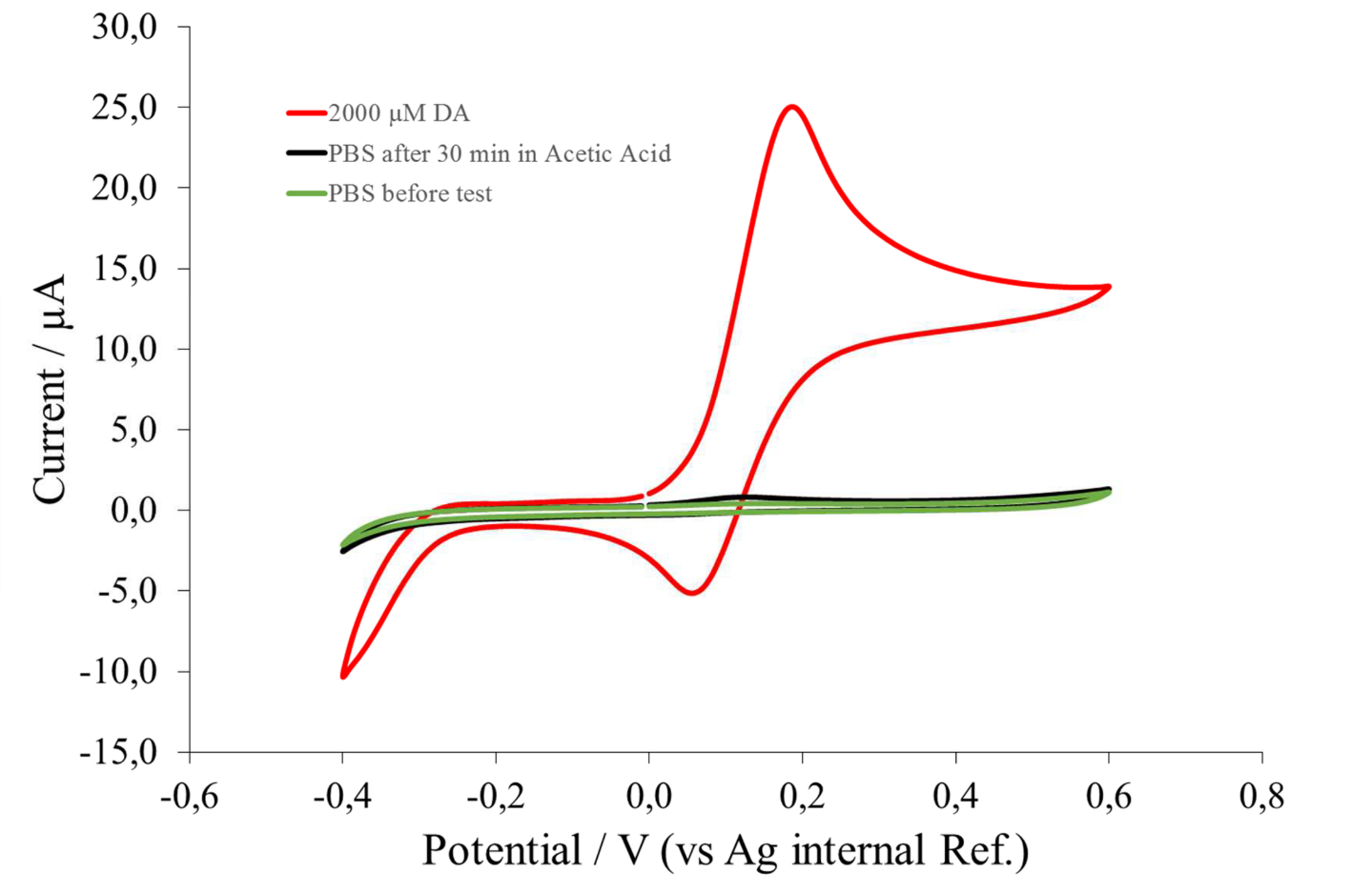


**Figure D.** Evaluation of the acetate solution washing on the retention of DA at the OxPPy-PBA film. The film was prepared from an acetonitrile water solution (1:4) containing 75 mM of Py and 1 mM of the dopant (PBA).





**Figure E.** Typical amperometric calibration, in PBS, for DA at the OxPPy-PBA film. The film was prepared from an acetonitrile water solution (1:4) containing 75 mM of Py and 1 mM of the dopant (PBA).


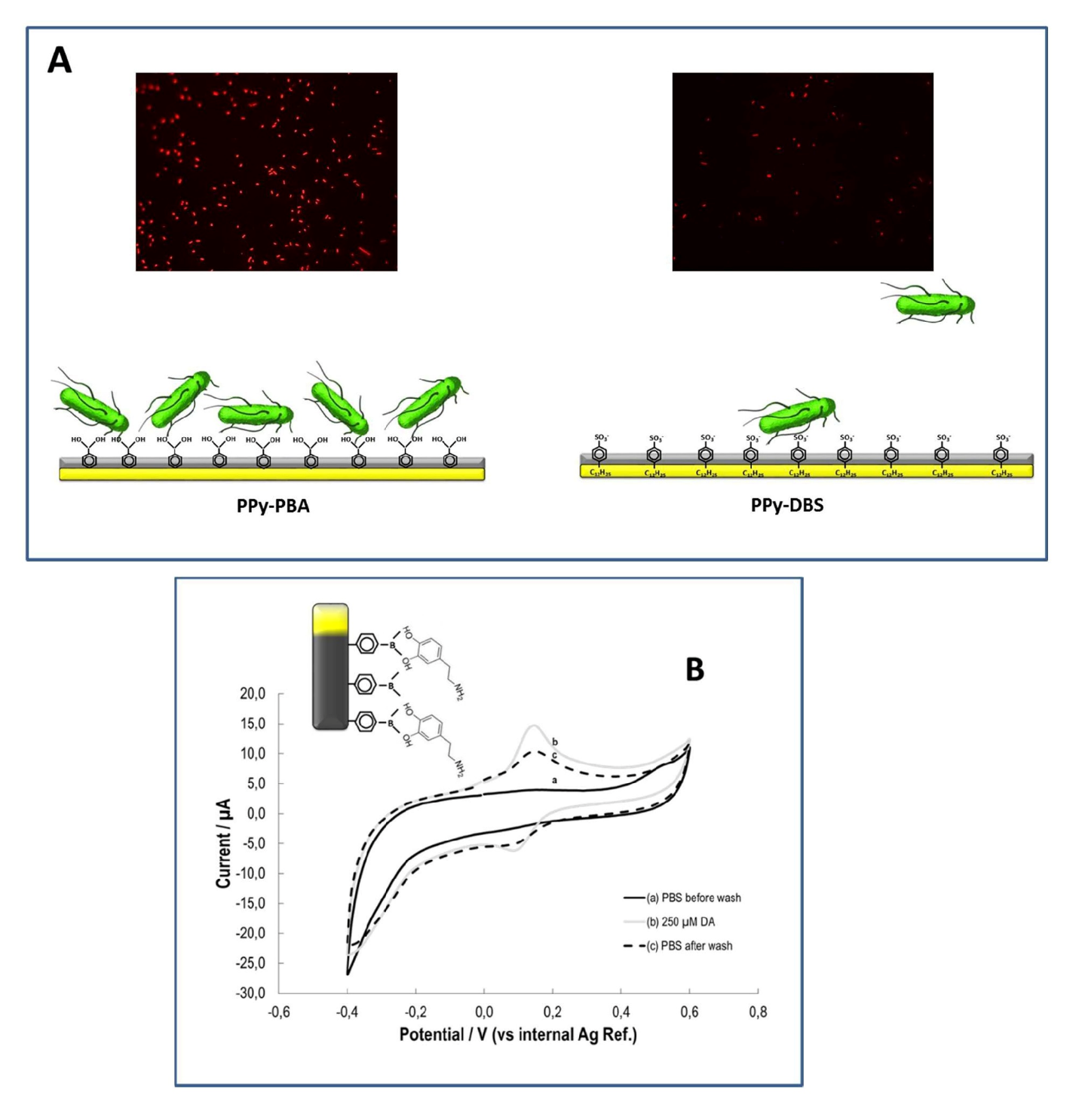


**Figure F.** Graphical abstract: Schematic representation of the use of boronic-modified dopant for the improved capture of bacteria (A) and of the concentration of dopamine (B).

| **Sensor Specification** | **Note** | **Analytical Performances** | **Application** | **Reference** |
| --- | --- | --- | --- | --- |
| AgNPs/PPy fiber composite.  Composite fabricated by in-situ electrochemical techniques onto GCE | Simultaneous detection of Ascorbic Acid (AA), Dopamine (DA) and Uric ACID (UA)  Detection method: Cyclic Voltammetry | Dynanic range:  AA: 10–580 μM, DA: 0.5–155 μM  UA: 2–100 μM,  Limit of detection  AA: 1.8 μM,  DA: 0.1 μM,  UA: 0.5 μM | Detection of DA in Dopamine injection | [[1](#_ENREF_1)] |
| AuNPs/Red. Graphene oxide/PPy composite.  Composite prepared in solution and drop casted onto GCE. | Selective detection of DA in the presence of AA and UA  Detection method: differential Pulse Voltammetry | Dynamic range:  0.1–5000 nM limit of detection:  18.29 pM. | Detection of DA in urine and serum | [[2](#_ENREF_2)] |
| OxPPy onto boron-doped diamond microfiber electrodes.  OxPPy film deposited by electrochemical techniques | Selective detection over AA and DOPAC (3,4-dihydroxyphenylacetic  acid).  Detection method:  Amperometry. | Dynamic range:  0.5 nM to 100 µM  Limit of detection:  0.01 nM | Laboratory solution | [[3](#_ENREF_3)] |
| PPy/reduced Graphene oxide composite.  Composite prepared by electrochemical techniques onto a GCE. | Selective detection of DA in the presence of AA and UA.  Detection method: differential Pulse Voltammetry | Dynamic range:  0.1–150 µM.  Limit of detection:  23 nM | Blood serum | [[4](#_ENREF_4)] |
| PPy (p-toluenesulfonate dopped)/reduced graphene oxide/Fe_3_O_4_ composite.  Composite prepared chemically and drop casted onto GCE | Selective detection of DA in the presence of AA and UA.  Detection method: Differential pulse voltammetry | Dynamic range:  7.0 nM–2.0 μM  Limit of detection:  2.33 nM | Detection in urine and blood serum | [[5](#_ENREF_5)] |
| PPy nano-sheeets/PtNPs composite.  Composite prepared by chemical process.  Sensor prepared by drop-casting composite onto GCE. | Selective detection of DA over AA and UA.  Detection method: Differential pulse voltammetry | Dynamic range:  0.01–400 µM  Limit of detection:  0.67 nM | Detection of DA in DA injections | [[6](#_ENREF_6)] |
| OxPPy/AuNPs composite  Composite prepared by electrodeposition onto a ZnOx nanotubes electrode. | Selective detection of DA in the presence of AA and UA.  Detection method:  Square Wave Voltammetry | Dynamic range:  25 nM to 2.5 µM  Limit of detection:  10 nM | Laboratory solution | [[7](#_ENREF_7)] |
| OxPPy (treated sodium dodecyl benzene sulfonate doped)/reduced graphene oxide composite  Composite prepared by electrochemical techniques onto GCE. | Selective detection of DA vs UA and AA  Detection method:  Square Wave Voltammetry | Dynamic range:  0.1-100 µM  Limit of detection:  20 nM | Spiked serum sample | [[8](#_ENREF_8)] |
| OxPPy (SDS doped)/reduced graphene oxide composite.  Composite deposited by electrochemical techniques onot GCE | Detection of DA in the presence of AA  Detection method:  Cyclic voltammetry | Dynamic range:  0.5 μM–10.0 µM  Limit of detection:   - 1. µM | Laboratory solution | [[9](#_ENREF_9)] |
| PPy film doped with sulfonated β-cyclodextrin  Film prepared by electrochemical technique onto Pt electrode. | Selective detection of DA in the presence of AA.  Detection method:  Amperometry | Dynamic range:  n.a.  Limit of detection:  3.2 µM | Laboratory solution | [[10](#_ENREF_10)] |
| OxPPy film doped with aszophloxine  Film prepared via electrochemical techniques onto Au electrode | Selective detection of DA and acetaminophen (AC).  Detection method:  Differential Pulse Voltammetry | Dynamic range:  DA: 0.1–30 µM  AC: 0.2–100 µM  Limit of detection:  DA: 50 nM  AC: 80 nM | Serum sample and pharmaceutical samples | [[11](#_ENREF_11)] |
| PPy tannic acid doped film  Film prepared by electrochemical techniques onto Au electrode | Selective detection of DA over AA.  Detection method:  Differential pulse voltammetry. | Dynamic range:  0 to 10 μM  Limit of detection:  0.3 µM | Laboratory solution | [[12](#_ENREF_12)] |
| OxPPy molecular imprinted polymer (MIP).  MIP deposited onto Pt microelectrode by electrochemical method | Selective detection of DA vs ascorbic acid, serotonin and 3,4-dihydroxyphenylacetic acid.  Detection method:  amperometry | Dynamic range:  10–100 nM  Limit of detection:  4.5 nM | In-vivo detection on rats following L-DOPA stimulation | [[13](#_ENREF_13)] |
| Poly(acrylamidophenylboronic acid) based molecular imprinted polymer (MIP).  MIP prepared by in-situ electrochemical polymerization onto Au electrode. | Selective detection of DA vs ascorbic acid, tyramine, homovanillic acid, and 3,4-dihydroxyphenylacetic acid.  Detection method:  Differential pulse voltammetry | Dynamic range:  50 nM to 2 µM  Detection limit:  Ca. 20 nM | Laboratory solutions | [[14](#_ENREF_14)] |
| OxPPy film doped with PBA  Film produced by electrochemical technique onto screen printed carbon electrode | Selective detection of DA over AA and acetaminophen (AC).  Detection method:  Amperometry | Dynamic range:  5 to 50 µM  Limit of detection:  4.1 µM | Laboratory solution | This work |

**Table A.** Comparison of proposed dopamine sensing surface with a selection of relevant literature.

**References.**

1. Ghanbari K, Hajheidari N (2015) Simultaneous electrochemical determination of dopamine, uric acid and ascorbic acid using silver nanoparticles deposited on polypyrrole nanofibers. Journal of Polymer Research 22.

2. Qian T, Yu C, Zhou X, Wu S, Shen J (2014) Au nanoparticles decorated polypyrrole/reduced graphene oxide hybrid sheets for ultrasensitive dopamine detection. Sensors and Actuators, B: Chemical 193: 759-763.

3. Olivia H, Sarada BV, Shin D, Rao TN, Fujishima A (2002) Selective amperometric detection of dopamine using OPPy-modified diamond microsensor system. Analyst 127: 1572-1575.

4. Si P, Chen H, Kannan P, Kim DH (2011) Selective and sensitive determination of dopamine by composites of polypyrrole and graphene modified electrodes. Analyst 136: 5134-5138.

5. Wang Y, Zhang Y, Hou C, Liu M (2016) Ultrasensitive electrochemical sensing of dopamine using reduced graphene oxide sheets decorated with p-toluenesulfonate-doped polypyrrole/Fe3O4 nanospheres. Microchimica Acta 183: 1145-1152.

6. Ghadimi H, Mahmoudian MR, Basirun WJ (2015) A sensitive dopamine biosensor based on ultra-thin polypyrrole nanosheets decorated with Pt nanoparticles. RSC Advances 5: 39366-39374.

7. Lin M (2015) A dopamine electrochemical sensor based on gold nanoparticles/over-oxidized polypyrrole nanotube composite arrays. RSC Advances 5: 9848-9851.

8. Daniel Arulraj A, Arunkumar A, Vijayan M, Balaji Viswanath K, Vasantha VS (2016) A simple route to Develop Highly porous Nano Polypyrrole/Reduced Graphene Oxide Composite film for Selective Determination of Dopamine. Electrochimica Acta 206: 77-85.

9. Zhuang Z, Li J, Xu R, Xiao D (2011) Electrochemical detection of dopamine in the presence of ascorbic acid using overoxidized polypyrrole/graphene modified electrodes. International Journal of Electrochemical Science 6: 2149-2161.

10. Harley CC, Rooney AD, Breslin CB (2010) The selective detection of dopamine at a polypyrrole film doped with sulfonated β-cyclodextrins. Sensors and Actuators, B: Chemical 150: 498-504.

11. Gholivand MB, Amiri M (2012) Simultaneous detection of dopamine and acetaminophen by modified gold electrode with polypyrrole/aszophloxine film. Journal of Electroanalytical Chemistry 676: 53-59.

12. Jiang L, Xie Q, Li Z, Li Y, Yao S (2005) A study on tannic acid-doped polypyrrole films on gold electrodes for selective electrochemical detection of dopamine. Sensors 5: 199-208.

13. Tsai TC, Han HZ, Cheng CC, Chen LC, Chang HC, et al. (2012) Modification of platinum microelectrode with molecularly imprinted over-oxidized polypyrrole for dopamine measurement in rat striatum. Sensors and Actuators, B: Chemical 171-172: 93-101.

14. Hong S, Lee LYS, So M-H, Wong K-Y (2013) A Dopamine Electrochemical Sensor Based on Molecularly Imprinted Poly(acrylamidophenylboronic acid) Film. Electroanalysis 25: 1085-1094.
